# Cell wall xyloglucan epitope modifications during flooding-induced root aerenchyma development in select cool-season legumes

**DOI:** 10.3389/fpls.2026.1873762

**Published:** 2026-07-08

**Authors:** Timothy J. Pegg, Robert L. Baker, Daniel K. Gladish

**Affiliations:** 1 Department of Biology, Midwestern State University, Wichita Falls, TX, United States; 2 Independent Researcher, Fort Collins, CO, United States; 3 Department of Biological Sciences, Miami University, Hamilton, OH, United States

**Keywords:** aerenchyma, cell wall, flooding, lysigenous, PCD, remodeling, root, xyloglucan

## Abstract

Characterizing the extracellular mechanisms plants use to adapt to water immersion could provide avenues for crop improvement in the face of periodic flooding. One such mechanism is hypoxia-induced aerenchyma formation. The formation of lysigenous aerenchyma occurs through programmed cell death (PCD) that may require the chemical modification of select polysaccharides in root cell walls. Currently, the precise mechanism of cell wall structural modification is not fully defined. To better characterize this mechanism, we investigated whether a relationship exists between modification of cell wall xyloglucans through removal of fucosyl functional groups and the formation of root aerenchyma in agriculturally relevant Fabaceae species. Characterization was conducted through the immunolabeling of specific xyloglucan epitopes within root cell walls during a 48-hour flooding time series. Immunolabeling results suggest progressive alterations in the accessibility or abundance of fucosylated and non-fucosylated xyloglucan epitopes in cell walls bordering developing aerenchyma cavities during PCD. Additionally, we performed an enzymatic pretreatment to remove select cell wall polymers prior to immunolabeling for xyloglucan, xylan and crystalline cellulose. These pretreatments demonstrate potential *in vitro* epitope masking constraints in root cell walls near developing aerenchyma. Our findings suggest that chemical modification to xyloglucans occurs in tandem with modification of select cell wall pectin epitopes. Our results elucidate previously uncharacterized cell wall carbohydrate remodeling during PCD leading to aerenchyma formation among legume species.

## 1 Introduction

As global climate change continues to alter weather patterns in the 21^st^ century, increased regional precipitation is predicted to intensify prolonged flooding events and significantly reduce crop yields due to low oxygen conditions (i.e. hypoxia) in roots (Hirabayashi et al., 2013; Striker and Colmer, 2016; Atanga and Tankpa, 2021). To minimize these losses, understanding plant adaptations to flooding is crucial for crop improvement efforts.

One type of flooding adaptation, aerenchyma formation in roots, results in large, air-filled channels or cavities in plant cortical or vascular tissues (Yamauchi et al., 2013; Takahashi et al., 2016). Aerenchyma allows plants to tolerate flooding-induced hypoxia by serving as an oxygen source within the cortex or stele (Yamauchi et al., 2013). The aerenchyma also helps maintain sufficient oxygen levels for cellular respiration by reducing the number of cells utilizing oxygen (Evans, 2004; Postma and Lynch, 2011; Yamauchi et al., 2013; Takahashi et al., 2016).

Aerenchyma may form through two general mechanisms: *schizogenous* mechanisms that utilize differential growth and cell separation to form internal tissue cavities without involving programmed cell death (PCD) or *lysigenous* mechanisms that involve PCD-mediated destructive remodeling and removal of cell wall components to form cavities. Lysigenous aerenchyma may be induced by environmental factors (Joshi and Kumar, 2012; Yamauchi and Nakazono, 2022) and involves the modification of the cell wall polysaccharide homogalacturonan in plants such as various cool-season legumes (Pegg et al., 2018, Pegg et al., 2020), *Oryza sativa* (rice) (Qu et al., 2016a), and *Zea mays* (maize) (Gunawardena et al., 2001b; Schneider et al., 2023). De-methyl-esterification during lysigenous aerenchyma formation enables enzyme access to linkages between residues of D-galacturonic acid that form the homogalacturonan backbone structure of pectin in the cell wall (Mohnen, 2008). Subsequent enzyme-mediated degradation of homogalacturonan in the cell walls leads to initiation and expansion of the aerenchyma cavities (Gunawardena et al., 2001b; Qu et al., 2016a).

The mechanisms directing modification of other major cell wall components such as hemicelluloses (e.g., xyloglucan) and cellulose during aerenchyma formation remains unclear. Observed cellulase (i.e., endo-β-glucanase) activity contributes to degradation of cell wall cellulose microfibrils during aerenchyma formation in *Zea mays* (Leite et al., 2017; He et al., 1994), *Helianthus annuus* (Kawase, 1979) and *Saccharum officinarum* (sugarcane) (Grandis et al., 2019). Xyloglucan and xylan linkages to cellulose may modulate cell wall degradation rates during aerenchyma formation (Leite et al., 2017). In addition, xyloglucan-cellulose interactions mediated by expansins have been documented during aerenchyma formation in *S. officinarum* (Grandis et al., 2019).

Alterations to the quantity and backbone structure of xyloglucan influence plant cell wall properties and numerous developmental events (Bou Daher et al., 2024). Changes in xyloglucan abundance have been observed to influence cell wall loosening in germinating *Arabidopsis thaliana* embryos (Sechet et al., 2016). Xyloglucan experiences chemical modification of the primary 1,4-linked β-D-glucose residues (Nishinari et al., 2007) through fucosylation and de-fucosylation of substituted xylose residues (Julian and Zabotina, 2022). The inhibition of fucosylation appears to inhibit *A. thaliana* root cell elongation (Dumont et al., 2015). It is possible that variations in specific fucose residues or shifts in cell wall matrix arrangement may modulate the susceptibility of xyloglucan to enzymatic degradation by exposing linkages between structural residues to hydrolytic enzyme activity, thereby acting consistent with a role in facilitating localized structural adjustment during the initiation of aerenchyma formation and continued lysigenous cavity growth.

The objective of our research was to evaluate the chemical alteration of cell wall xyloglucan during aerenchyma formation of several cool-season legumes. The authors hypothesized that shifts in the spatial and temporal distribution of specific xyloglucan epitopes would be associated with a potential xyloglucan structural remodeling pathway. This adaptive variation in xyloglucan epitope accessibility or apparent abundance may share features with previously described cell wall modification functions of homogalacturonan de-methyl-esterification (Wolf et al., 2009; Pegg et al., 2025), thereby correlating with the localized matrix modifications observed during root aerenchyma development. In support of the hypothesis, alterations in the spatial and temporal localization of specific xyloglucan epitopes were observed during flooding-induced aerenchyma formation in *Cicer arietinum*, *Pisum sativum* and *Phaseolus coccineus.* We present evidence of altered fucosylated and non-fucosylated xyloglucan epitope accessibility in cell walls during programmed cell death that is concurrent with standard aerenchyma cavity expansion. Availability of additional cell wall polymers that surround xyloglucan, such as select cellulose and pectin epitopes, may also be altered, thereby permitting potential *in situ* hydrolytic enzymatic cleavage associated with the progression of lysigenous root aerenchyma formation (Yamauchi and Nakazono, 2022). Our findings suggest that chemical modification of the xyloglucan occurs in tandem with modification of select pectin epitopes (Pegg et al., 2018, Pegg et al., 2020) to accommodate normal aerenchyma development.

## 2 Results

### 2.1 Cell wall and middle lamella degrade during aerenchyma formation

**Figure 1:**
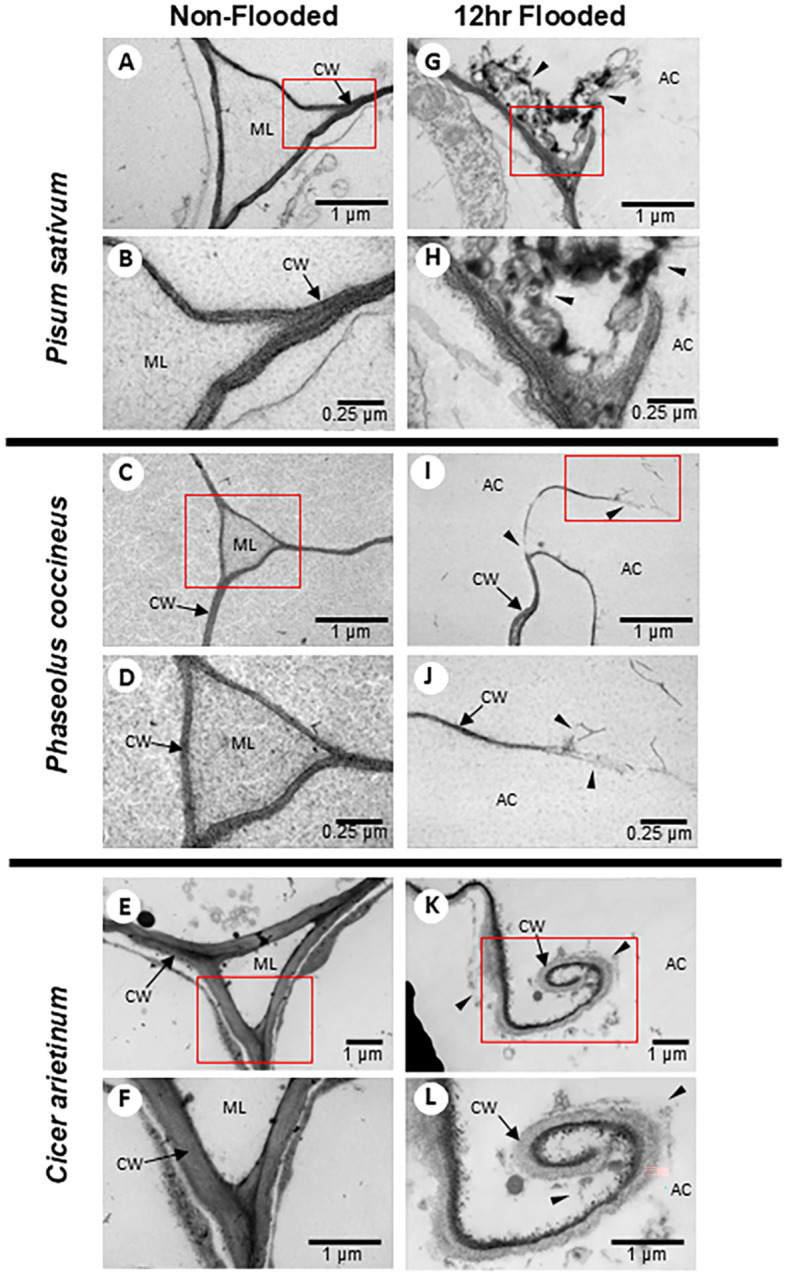
Adjacent cellular layers alteration during aerenchyma formation in legume roots. Root cross-sections were stained with uranyl acetate, osmium tetroxide and ruthenium red for **(A–F)** non-flooded samples at two magnifications for **(A, B)** *Pisum sativum*, **(C, D)** *Phaseolus coccineus*, and **(E, F)** *Cicer arietinum*. Opposite column displays comparison with **(G–L)** roots flooded for 12 hours at two magnifications for **(G, H)** *P. sativum*, **(I, J)** *P. coccineus*, and **(K, L)** *C. arietinum*. ML = middle lamella, AC = aerenchyma cavity, CW = cell wall. Red boxes show magnified areas. Arrows and brackets indicate areas suggesting degradation of cell wall components.

Degradation of cell walls in *Cicer arietinum*, *Phaseolus coccineus*, and *Pisum sativum* were examined via transmission electron micrographs of flooded and non-flooded treatments (**Figure 1**). Transmission electron microscopy was deployed strictly as a descriptive tool to document structural alterations during early-stage matrix modification (0 and 12 hours). Unlike non-flooded samples (**Figures 1A–F**), 12-hour flooding samples (**Figures 1G–L**) demonstrated degradation of cell walls and organelles leading to the formation of aggregates near aerenchyma cavities in each species. Dense labeling patterns from ruthenium red and osmium tetroxide staining in 12-hour flooded samples suggested these aggregations were comprised of pectic polysaccharides and phospholipids from plasma membranes, respectively, based on binding specificities described in previous research (McFarlane et al., 2014; Lionetti, 2015; Kiyoto et al., 2022). Compared to *P. sativum* and *P. cocinneus* (**Figures 1A-D, G-J**), the primary cell walls in C. *arietinum* (**Figures 1E, F, K, L**) appeared to present greater labeling density. The presence of additional cell wall polymers and surface area in the larger cell walls of C. *arietinium* (**Figures 1E, F, K, L**) for TEM stain binding sites may explain this discrepancy. Curling and fragmentation of cell walls (**Figures 1G–L**) were observed across biological replicates and imply alteration of additional cell wall polysaccharides and oligosaccharides alongside homogalacturonan. Follow-up fluorescent immunolabeling experiments investigated the potential role of xyloglucans in generating the observed aggregations of degraded cellular components near cell walls bordering aerenchyma.

### 2.2 Immunolabeling suggests chemical alteration of xyloglucan during root aerenchyma formation

Evaluation of cell wall oligosaccharides alteration during aerenchyma formation was conducted through the labeling of root cross-sections of several legume species (*C. arietinum*, *P. sativum* and *P. coccineus*), with monoclonal antibodies targeting fucosylated xyloglucan (CCRC-M1) and non-fucosylated xyloglucan (CCRC-M100).

**Figure 2:**
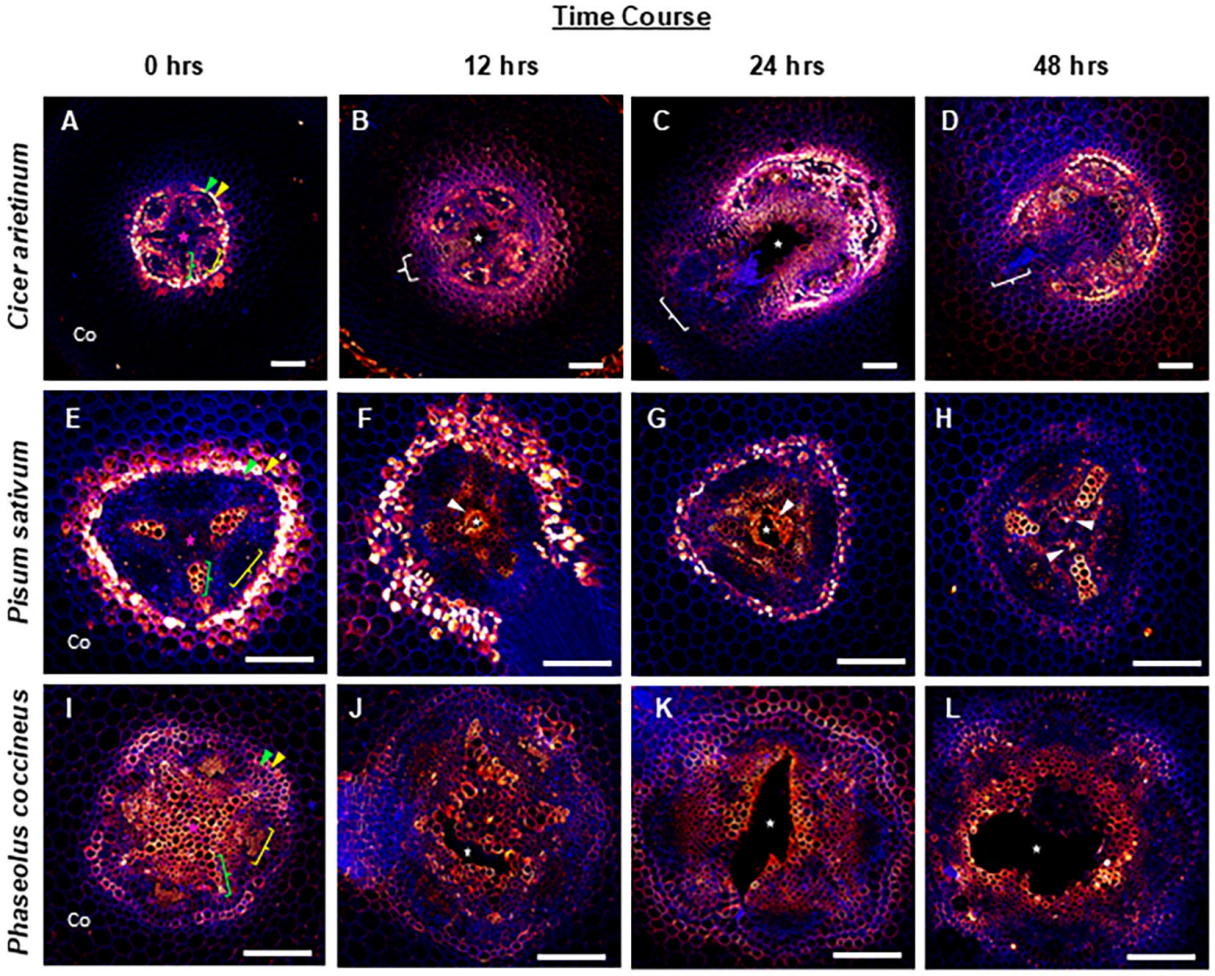
Absence of fucosylated xyloglucan (CCRC-M1) labeling observed during aerenchyma formation in *Cicer arietinum*. Micrographs display localization of CCRC-M1 antibody labeling for non-flooded and 12-48-hour flooded roots for **(A–D)** *Cicer arietinum*, **(E–H)** *Pisum sativum* and **(I–L)** *Phaseolus coccineus*. Micrographs indicate the endodermis (yellow wedge), pericycle (green wedge), xylem (green bracket), phloem (yellow bracket), and central parenchyma (pink star) within root cross sections. Cells prominently labeled with antibodies are indicated with white wedges, while loss of labeling is indicated with white brackets. Aerenchyma cavities are indicated with white stars. Antibody labeling and aldehyde-induced fluorescence are assigned red/yellow and blue false-colors, respectively. Co = cortex. Scale bars = 100 µm.

Our immunolabeling results using CCRC-M1 provide a qualitative assessment of spatial and temporal evidence supporting the hypothesis that selective xyloglucan remodeling correlates with the broader structural alterations observed during normal aerenchyma development (**Figure 2**). A notable lack of CCRC-M1 antibody binding was observed within *Cicer arietinum* cortical, pericycle, and endodermis cells preceding the expansion direction of the developing aerenchyma cavities at the 12–48-hour flooding time points (**Figures 2B–D**). This reduced detection of the fucosylated xyloglucan epitope implies a localized alteration in root cell wall structural configuration or masking profile, rather than serving as direct biochemical proof of *in vivo* enzymatic de-fucosylation. Similar to the well-characterized role of homogalacturonan de-methyl-esterification (Wolf et al., 2009; Pegg et al., 2025), this type of hemicellulose modification may alter cell wall porosity or expose the xyloglucan backbone to subsequent enzymatic cleavage. These observations are consistent with a potential role in facilitating matrix loosening during structural reorganization and aerenchyma cavity expansion.

In contrast, CCRC-M1 binding patterns observed in *Pisum sativum* imply a different functional manifestation of this putative remodeling mechanism. In our data, prominent CCRC-M1 binding was retained and concentrated within young metaxylem cells adjacent to the expanding aerenchyma at 12–24 hours of flooding (**Figures 2F–H**). This localized labeling suggests that while the surrounding cortical cells may undergo clearing, these critical vascular elements maintain fucosylated xyloglucan networks, potentially functioning in tandem with select pectin epitopes (Pegg et al., 2018, Pegg et al., 2020) to reinforce the root vascular cylinder against the mechanical stresses and altered turgor pressures induced by surrounding cortical lysis.

The stability of the CCRC-M1 labeling pattern across all time points in *Phaseolus coccineus* implies that shifts in fucosylated xyloglucan network density or distribution may not be a universal prerequisite for cortical remodeling across all cool-season legumes (**Figures 2I–L**). This suggests that *P. coccineus* possesses potential alternative cell wall remodeling pathways involving distinct cellulose or pectin matrix alterations to mediate structural responses to flooding. Collectively, the CRCC-M1 patterns observed between *Cicer arietinum*, *P. sativum*, and *P. coccineus* indicate that chemical modifications altering the availability of fucosylated xyloglucan epitopes may be dynamically coordinated with surrounding cell wall matrix polymers to either permit or restrict cell wall degradation during aerenchyma formation. These divergent, species-specific immunolocalization signatures highlight three distinct micro-architectural differences among the studied taxa. First, an abrupt decline in cortical tissue labeling signal in *C. arietinum* representing a highly plastic structural response in those cell walls. Second, the preservation of localized antibody binding in vascular tissue *P. sativum* suggests a similarly localized mechanical enforcement mechanism in those cell walls. Finally, the observed antibody labeling signal in *P. coccineus* indicates uniform cell wall matrix stability throughout the root tissues of samples subjected to flooding that is fundamentally distinct from proposed localized cell wall alterations of *P. sativum.*

**Figure 3:**
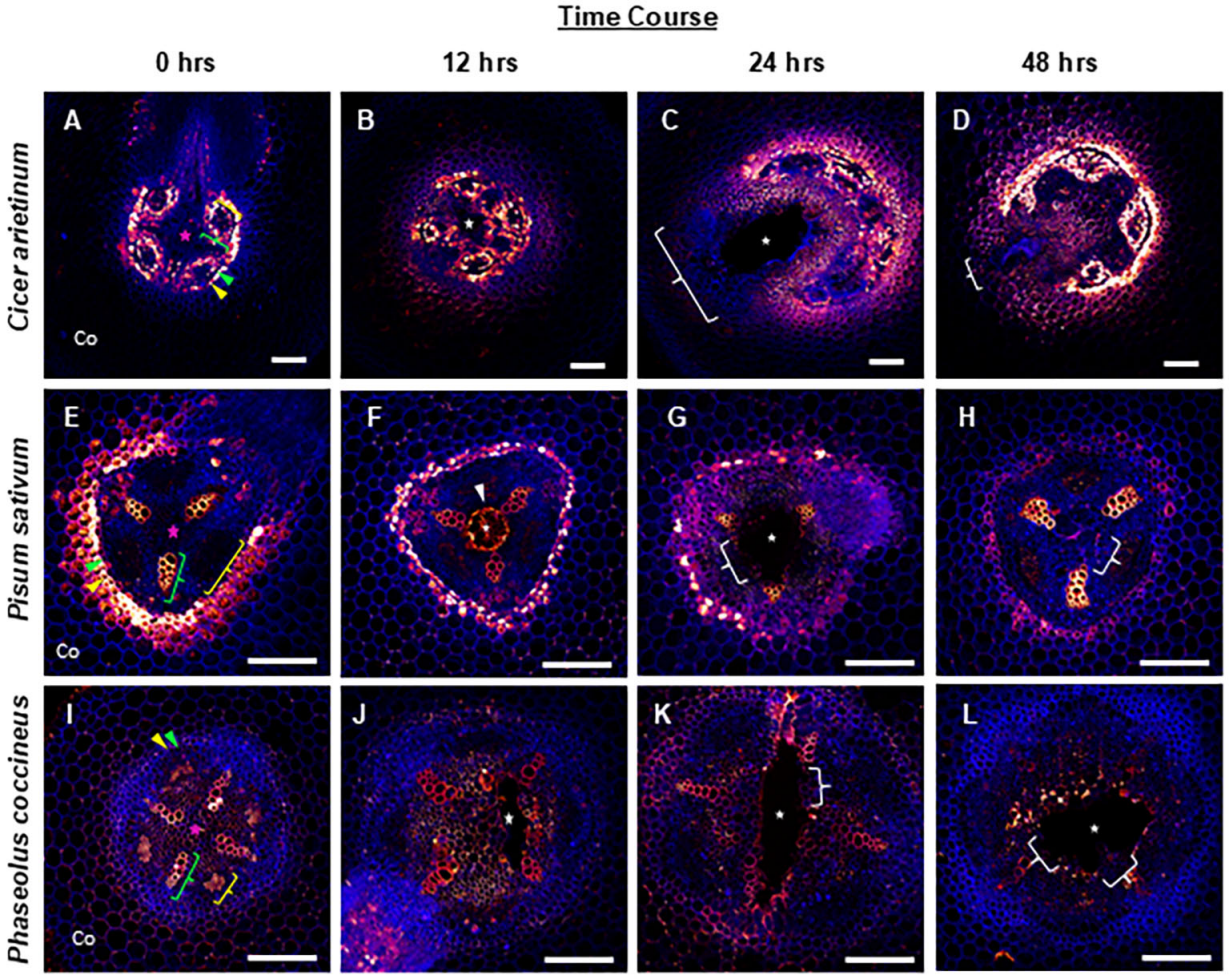
Absence of non-fucosylated xyloglucan (CCRC-M100) labeling observed in later stages of aerenchyma development in legume roots. Micrographs display localization of CCRC-M100 antibody labeling for non-flooded and 12-48-hour flooded roots for **(A–D)** *Cicer arietinum*, **(E–H)** *Pisum sativum* and **(I–L)** *Phaseolus coccineus.* Micrographs indicate the endodermis (yellow wedge), pericycle (green wedge), xylem (green bracket), phloem (yellow bracket), and central parenchyma (pink star) within root cross sections. Cells that are prominently labeled with antibodies are indicated with white wedges, while loss of labeling is indicated with white brackets. Aerenchyma cavities are indicated with white stars. Antibody labeling and aldehyde-induced fluorescence are assigned red/yellow and blue false-colors, respectively. Co = cortex. Scale bars = 100 µm.

CCRC-M100 antibody binding, which targets non-fucosylated xyloglucan, exhibited distinct, time-dependent patterns of absence across the roots of all three legume species during flooding (**Figure 3**). In *C. arietinum*, consistent CCRC-M100 labeling was initially localized within the cortical cells, endodermis, and pericycle at both the 0-hour (control) and 12-hour flooding timepoints (**Figures 3A, B**). Conversely, both *P. coccineus* and *P. sativum* displayed an absence of antibody labeling during these early 0-hour and 12-hour periods (**Figures 3E, F, I, J**). During later stages of flooding (24 and 48 hours), *C. arietinum* experienced a complete absence of localized binding in the cell walls directly preceding the expanding aerenchyma (**Figures 3C, D**), indicating an alteration in epitope exposure or structural shielding of non-fucosylated xyloglucan domains. A similar absence of labeling was maintained adjacent to the late-stage aerenchyma cavities in *P. coccineus* and *P. sativum* (**Figures 3G, H, K, L**). Collectively, these antibody labeling patterns suggest that modifying the visibility of non-fucosylated xyloglucan epitopes within cell walls adjacent to forming aerenchyma are characteristic morphological features of late-stage structural modifications across all three legume species.

### 2.3 Enzyme pretreatments suggest that unmasking of xyloglucan may occur during aerenchyma formation

**Figure 4:**
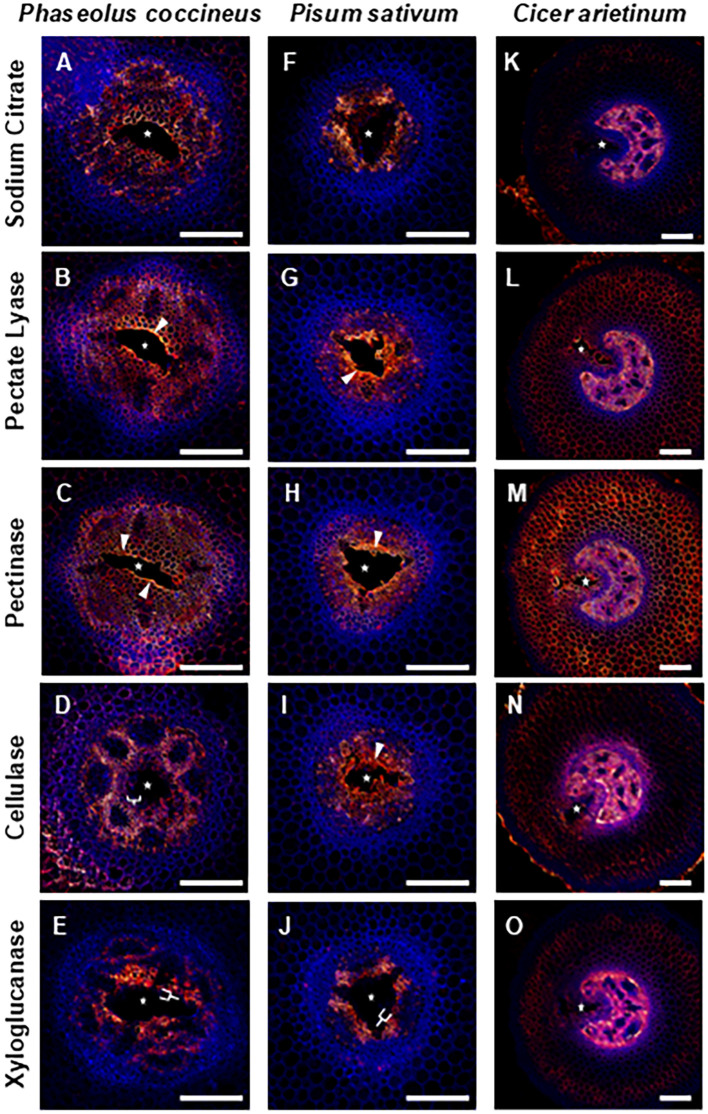
Unmasking of fucosylated xyloglucan (CCRC-M1) in 24-hour flooded *Pisum sativum* and *Cicer arietinum* roots displaying aerenchyma formation. The labeling pattern of CCRC-M1 in **(A–E)** *Phaseolus coccineus*, **(F–J)** *Pisum sativum* and **(K–O)** *Cicer arietinum* root cross sections displayed after samples are treated with **(A, F, K)** sodium citrate, **(B, G, L)** pectate lyase, **(C, H, M)** pectinase, **(D, I, N)** cellulase, and **(E, J, O)** xyloglucanase enzyme solutions. Antibody labeling and aldehyde-induced fluorescence are assigned red/yellow and blue false-colors, respectively. Cells that are prominently labeled with antibodies are indicated with white wedges, while loss of labeling is indicated with white brackets. White stars and wedges indicate aerenchyma cavities. Scale bars = 100 µm.

**Figure 5:**
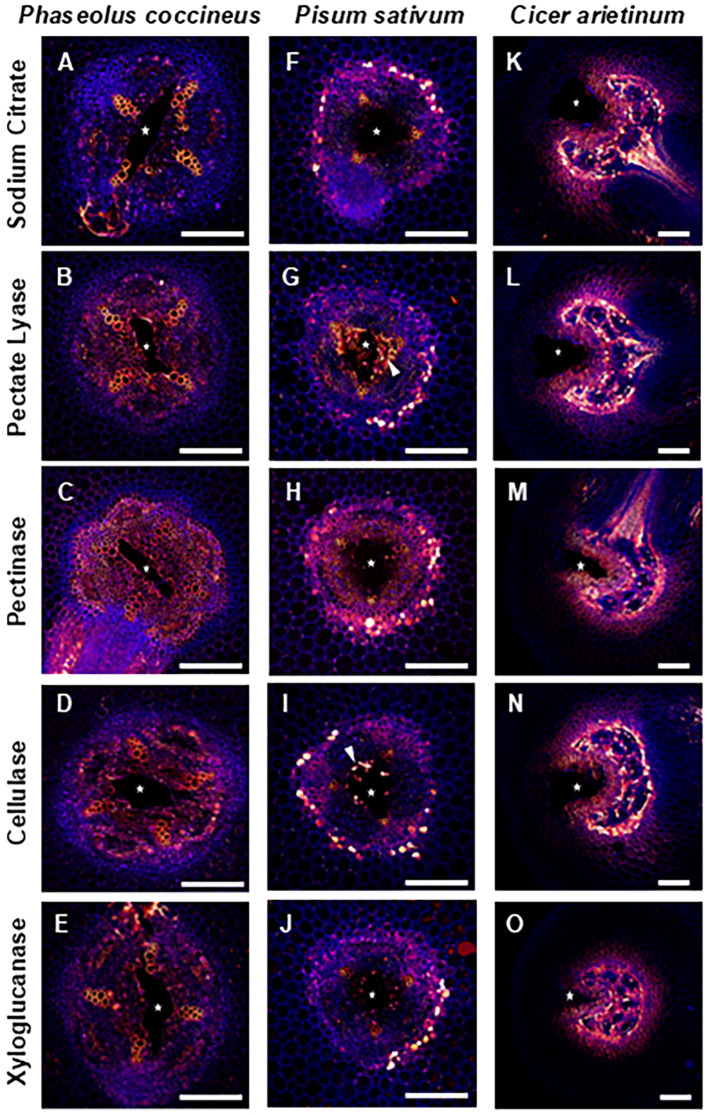
Unmasking of non-fucosylated xyloglucan (CCRC-M100) in 24-hour flooded *Phaseolus coccineus* and *Pisum sativum* roots displaying aerenchyma formation. The labeling pattern of CCRC-M100 in **(A–E)** *Phaseolus coccineus*, **(F–J)** *Pisum sativum* and **(K–O)** *Cicer arietinum* root cross sections displayed after samples were treated with **(A, F, K)** sodium citrate, **(B, G, L)** pectate lyase, **(C, H, M)** pectinase, **(D, I, N)** cellulase, and **(E, J, O) xyloglucanase** enzyme solutions. Antibody labeling and aldehyde-induced fluorescence are assigned red/yellow and blue false-colors, respectively. Cells that are prominently labeled with antibodies are indicated with white wedges, while loss of labeling is indicated with white brackets. White stars and wedges indicate aerenchyma cavities. Scale bars = 100 µm.

To evaluate the potential masking of xyloglucan by other cell wall polysaccharides during root aerenchyma formation, we utilized targeted exogenous enzyme pretreatments to sequentially remove matrix components prior to antibody labeling. Interpretations of enzyme pretreatment effects were based on qualitative comparisons of labeling patterns observed consistently across biological replicates. Root tissue sections were pretreated with specific glycan-degrading enzymes to determine whether the apparent loss of fucosylated xyloglucan (CCRC-M1) and non-fucosylated xyloglucan (CCRC-M100) epitopes was due to cell wall polymer degradation or steric hindrance by overlapping wall components disrupting antibody binding (**Figures 4**, **5**). Changes in antibody labeling patterns following these extractions were evaluated to assess epitope accessibility and determine if matrix stripping unmasked hidden xyloglucan domains.

Pretreatment of CCRC-M1 antibody-labeled roots revealed distinct localization trends for fucosylated xyloglucans across *P. coccineus, P. sativum*, and *C. arietinum*. Compared to sodium citrate controls (**Figures 4A, F, K**), pretreatments with pectate lyase and pectinase resulted in enhanced epitope accessibility in cell layers adjacent to aerenchyma cavities in all three species (**Figures 4B, C, G, H, L, M**). Pretreatment with cellulase demonstrated variable antibody binding patterns across the legumes with a loss of antibody labeling occurred in cells bordering the aerenchyma of *P. coccineus* (**Figure 4D**), whereas increased labeling consistency or intensity was observed in cells adjacent to the aerenchyma of *P. sativum* (**Figure 4I**) and within the cortical cells of *C. arietinum* (**Figure 4N**). Pretreatment with xyloglucanase appeared to alter epitope availability in cells immediately adjacent to the aerenchyma in P. coccineus and P. sativum, resulting in diminished primary antibody binding (**Figures 4E, J**). By contrast, xyloglucanase pretreatment increased epitope accessibility in the cortex of *C. arietinum* (**Figure 4O**) compared to the control (**Figure 4K**), mirroring the pattern observed following cellulase pretreatment (**Figure 4N**). These results indicate that the pectin backbone or associated pectin residues may physically restrict antibody access to fucosylated xyloglucan in intact primary cell walls. Enzymatic removal of cellulose similarly enhances *in vitro* epitope accessibility in *C. arietinum* and *P. sativum*, but appears to compromise epitope retention or availability in *P. coccineus*. These observations suggest that *P. coccineus* cell walls may feature distinct xyloglucan-cellulose structural interactions compared to those of *C. arietinum* and *P. sativum*.

Qualitative, visual evaluation of reproducible CCRC-M100 antibody-labeling patterns across biological replicates unveiled non-fucosylated xyloglucan localization patterns conserved in both *P. sativum* and *P. coccineus*. When compared to sodium citrate pretreatments for CCRC-M100 antibody labeling (**Figures 5A, F**), pretreatments with pectate lyase, pectinase and cellulase revealed visually distinct alterations in antibody binding consistency in cells adjacent to aerenchyma cavities in *P. coccineus* and *P. sativum* (**Figures 5B–D, G-I**). By contrast, pretreatment with xyloglucanase (**Figures 5E, J**) resulted in an apparent decrease in antibody binding in *P. coccineus* relative to *P. sativum*. Notably, CCRC-M100 labeling consistency remained visibly unchanged across all treatments in *C. arietinum* (**Figures 5K–O**).

Based on these qualitative micrographs, the data suggests that extraction of pectin and cellulose enhances *in vitro* epitope accessibility for non-fucosylated xyloglucan in *P. coccineus* and *P. sativum*. Conversely, non-fucosylated xyloglucan in *C. arietinum* root cell walls preceding the expanding aerenchyma is either absent or remains inaccessible despite these enzyme pretreatments. While quantitative measurements were not performed, these descriptive observations collectively suggest that the composition or structural organization of *C. arietinum* root cell walls may differ from those found in *P. sativum* and *P. coccineus*.

## 3 Discussion

Our study demonstrated that fucosylated and non-fucosylated xyloglucan localization patterns correlate closely with the geometric changes driving lysigenous aerenchyma development in *Pisum sativum*, *Phaseolus coccineus*, and *Cicer arietinum*. We present immunolocalization data suggesting that distinct chemical epitopes of xyloglucan are progressively modified within the cell walls bordering expanding aerenchyma air spaces. Furthermore, exogenous enzyme pretreatments indicate that surrounding cell wall matrix polymers – specifically pectins and cellulose – physically restrict xyloglucan epitope accessibility in a species-specific manner. Derived from our qualitative, spatially resolved immunolocalization patterns, these findings suggest that the structural modification of hemicellulosic xyloglucans occurs in tandem with targeted alterations to select pectin epitopes (Pegg et al., 2018, Pegg et al., 2020) to correspond with cell wall loosening during lysigenous aerenchyma development.

We observed alterations to cell wall structure within the parenchyma tissues bordering developing root aerenchyma, which is consistent with previous research in legumes (Pegg et al., 2020; Sarkar et al., 2008) and *Zea mays* (Gunawardena et al., 2001a). Flooding-induced aerenchyma formation exhibited marked degradation of the middle lamella and cell walls during early stages of cavity development in all three legume species (**Figure 1**). TEM micrographs and fluorescent antibody labeling pattern alterations observed during non-flooding and flooding treatments suggest alteration of multiple cell wall oligosaccharides and polysaccharides (**Figures 1**-**3**, **Supplementary Figures 1**, 2) lysigenous aerenchyma development in *C. arietinum*, *P. coccineus*, and *P. sativum*.

Immunolabeling assays were utilized to examine select cell wall modifications associated with cell wall polymer degradation near the forming aerenchyma cavity. Our data suggests that lysigenous aerenchyma development is accompanied by the remodeling of specific hemicellulose components in cool-season legumes, as the apparent abundance of both non-fucosylated and fucosylated xyloglucan diminished in cell walls adjacent to the aerenchyma over increasing flooding durations (**Figures 2**, **3**). Altered antibody labeling patterns resemble previous observations evaluating alterations to homogalacturonan epitopes via de-methyl-esterification (DME) during aerenchyma formation (Pegg et al., 2020; Sarkar et al., 2008). While the precise biochemical sequence remains to be determined, the concurrent loss of distinct xyloglucan epitopes suggests that shifts in xyloglucan structure or side-chain composition occur alongside structural alterations to the pectin matrix during cell wall remodeling. In other developmental contexts, such as fruit ripening, leaf abscission, and lateral root emergence, coordinated modifications to both pectin and hemicellulose side-chains help regulate cell wall loosening and localized wall remodeling (Hyodo et al., 2013; Lashbrook and Cai, 2008; Vilches-Barro and Maizel, 2015; Ma et al., 2021). Previous work in *Arabidopsis thaliana* also supports xyloglucan modification as a primary driver of cell wall loosening in tissues such as seed endosperm (Sechet et al., 2016). Our findings extend this concept to flooding-induced lysigenous aerenchyma formation, suggesting that modulation of xyloglucan chemistry within the cell wall matrix may represent a key component of the structural remodeling associated with accommodating hypoxia-induced air cavity development.

The observed CCRC-M1 antibody-labeling patterns (**Figure 2**) may reflect species-specific differences in developmental timing, root anatomy, and the organization of the cell wall cellulose-xyloglucan-pectin matrix during flooding responses. In *C. arietinum*, the poor labeling of fucosylated xyloglucan epitopes may indicate either an earlier onset of wall remodeling in cortical tissues or a more rapid transition toward epitope masking or loss in cells adjacent to the developing aerenchyma. By contrast, the persistence of localized CCRC-M1 signal in *P. sativum* metaxylem suggests that xyloglucan modification may be spatially restricted to specific tissues rather than occurring uniformly across the root. The comparatively stable antibody labeling pattern in *P. coccineus* may reflect a different strategy of wall remodeling, in which xyloglucan epitopes remain more accessible or are modified more slowly during flooding. These differences could also arise from species-specific variation in root tissue architecture, including the relative development of cortex, endodermis, and vascular cylinder, as well as differences in the degree to which xyloglucan is integrated with cellulose microfibrils and pectin-rich wall domains. Because our study was designed as a qualitative, rather than quantitative, spatial analysis time-course across developmentally-matched root tissues, we cannot conclusively confirm that observed patterns primarily reflect differences in developmental stage, cell wall composition, or flooding tolerance strategy. Nonetheless, the contrasting labeling signatures across the three legume taxa are consistent with the idea that aerenchyma-associated wall remodeling is coordinated in a species-dependent manner and may be linked to distinct structural and physiological strategies for coping with flooding-induced hypoxic stress.

We performed enzyme pretreatment assays to remove select cell wall components that surround non-fucosylated and fucosylated xyloglucan. The objective of these assays was to evaluate the potential masking of non-fucosylated or fucosylated xyloglucan through observation of antibody labeling pattern alterations after the extraction of pectin, cellulose, and other matrix polymers from root cell walls. While these treatments investigate the structural accessibility of the cell wall, they demonstrate potential *in vitro* epitope masking constraints rather than definitively mapping the temporal sequence of remodeling events occurring *in vivo* within living root tissue.

Our research suggests that pectin and cellulose (**Figures 4**, **5**; **Supplementary Figure 3**) physically restrict antibody access to both non-fucosylated and fucosylated xyloglucan chemical domains. Such binding interactions within primary cell walls may cause steric hindrance, thereby blocking antibody access to target epitope binding sites (Marcus et al., 2008). The existence of these masking effects suggests that xyloglucan modification may occur across a broader region of the root central parenchyma than was initially apparent in un-extracted immunolabeling experiments (**Figure 2**, **3**). This interpretation is supported by results from the pectate lyase, pectinase, and cellulase pretreatments (**Figures 4B, C, G–I, L-N**, **5B-C, G-I**), where antibody labeling within the central parenchyma, endodermis, and pericycle was more pronounced compared to untreated control samples (**Figures 4A, F, K**, **5A, F**). These results imply that the structural modification of xyloglucan may proceed independently from the removal of other cell wall components during aerenchyma formation. Additionally, the timing and order of cell wall alterations during aerenchyma development may strongly depend on the specific plant taxa under evaluation.

Specifically, xyloglucan and cellulose may interact during aerenchyma formation. The labeling patterns of cellulose (**Supplementary Figure 2**, **3**) and xyloglucan (**Figures 2**-**4**) hint at their co-localization within the matrix. Cellulose and xyloglucan networks function as the primary structural support components in plant primary cell walls (Pauly et al., 1999; Cosgrove, 2001). Previous research established that cellulose-xyloglucan aggregations form in cell walls bordering aerenchyma in *S. officinarum* roots (Grandis et al., 2019), potentially serving to stabilize aerenchyma boundaries. A similar phenomenon occurs in cells bordering the outer layer of the pea root cap in *P. sativum* (Ropitaux et al., 2019). Future antibody co-localization experiments may clarify whether similar structures are degraded during early-stage aerenchyma formation or retained to reinforce late-stage legume aerenchyma boundaries.

In addition to the enzymatic regulation of lysigenous aerenchyma formation and programmed cell death, exploring the gene expression patterns that influence xyloglucan remodeling is another promising avenue for further research. While direct transcriptomic profiling or functional gene validations were outside the scope of this spatial-mapping study, previous research in *Arabidopsis thaliana* suggests genes encoding expansins (Cho and Cosgrove, 2002) and xyloglucan endotransglucosylases/hydrolases (Kurasawa et al., 2009) may alter xyloglucan chemical structure in primary cell walls (Cosgrove, 2001; Purugganan et al., 1997). Furthermore, specific fucosyltransferase (FUTase) genes direct the addition of fucose residues to galactose residues on xyloglucan side chains (Perrin et al., 2003), thereby modulating the degree of wall polymer fucosylation. Similar to research in *Zea mays* (Rajhi et al., 2011), experiments incorporating laser-capture microdissection of cells adjacent to the developing legume root aerenchyma followed by RNA sequencing could result in identification of *A. thaliana* xyloglucan orthologs representative of these remodeling gene families. Additionally, transcriptomic analysis, such as those conducted on bermudagrass (*Cynodon dactylon*) subjected to flooding stress, may indicate the enhanced presence of xyloglucan remodeling enzymes during aerenchyma formation (Yuan et al., 2021). Alongside enzyme assays to measure endotransglucosylase/hydrolases, FUTase, and xylanase activity, the temporal and spatial regulation of cell wall degradation and xyloglucan fucosylation may be revealed in future research projects.

In summary, our study investigated the role of cell wall xyloglucan during flooding-induced vascular aerenchyma formation of the cool-season legumes *P. sativum*, *P. coccineus* and *C. arietinum*. Our experimental results suggest that cell wall xyloglucans are modified in an analogous manner as homogalacturonan epitopes during flooding-induced aerenchyma formation of cool-season legumes (Pegg et al., 2020). Namely, variations in the accessibility or configuration of specific structural epitopes occur in the primary walls of cells adjacent to developing aerenchyma and appear to be associated with progressive aerenchyma cavity development. Additionally, our enzyme pretreatment assay suggests that cell wall polymers, such as the cellulose and xyloglucan comprising an interacting wall matrix, may also display complex spatial masking combinations concurrent with aerenchyma air space development. We propose that these metabolic events within the primary cell wall contribute to destabilization of the cell wall matrix and serve as a mechanism by which programmed cell death removes cells to enlarge the aerenchyma. An improved understanding of the cell wall dynamics investigated in our study could enable crop improvement focused on modulating aerenchyma develop in roots subjected to flooding-induced hypoxic soil conditions. Increases in aerenchyma development rate, size, or inducing aerenchyma formation in plants that do not normally develop this morphological feature, may lead to increased root flooding tolerance and substantial yield improvement of crops within agricultural regions exposed to prolonged flooding conditions.

## 4 Methods

### 4.1 Seedling growth and flooding treatment

Seedlings were grown according to Gladish and Niki (2000). For each species, 40 seeds (*Pisum sativum* and *Cicer arietinum*), or 20 seeds (*Phaseolus coccineus*), were sown, per beaker, into 2 L beakers filled with sterile, super-coarse vermiculite (Perlite Vermiculite Packaging Industries, Inc., USA), moistened with deionized water, and covered with aluminum foil. Beakers were placed into 25 °C growth chambers for 5 d in complete darkness to initiate root growth. Five technical replicates for each flooding treatment (0, 12, 24, and 48 h water immersion) were created using a separate 2 L beaker for each replicate.

To perform treatments, four sets of beakers containing growing seedling roots (0, 12, 24, and 48 h water immersion) were removed from growth chambers and placed under a laminar flow hood, where three of the four beaker sets (12, 24, and 48 h time points) were filled with sterile deionized water to the surface of the vermiculite. Three non-flooded beakers containing seedlings were harvested immediately for the 0-hour timepoint. The remaining beakers were returned to the 25°C growth chambers and removed at either 12, 24, or 48 hours after flooding to be harvested for sectioning.

### 4.2 Sectioning, fixation and embedding

Sectioning, fixation, and embedding were performed according to Pegg et al. (2020). Briefly, a minimum of 5 root segments were harvested from unique, individual plants of each species per timepoint. This procedure was repeated three times to ensure three biological replicates for each species per timepoint. Segments were cut with razor blades (Electron Microscopy Services, USA) from either 1.5–5 cm (*P. sativum* and *P. coccineus*) or 3–7 cm (*C. arietinum*) away from root tips after the presence of aerenchyma cavities were confirmed under a dissection microscope in the later timepoints. As all timepoints were harvested at the same distance from the growing root apex, they were at the same approximate developmental stage and the 0-hour timepoint serves as a control for all three flooding timepoints. Segments were fixed in 1% paraformaldehyde and 2% glutaraldehyde solution in deionized water in 0.1M sodium cacodylate buffer, pH 7.4, at 5 °C. Segments were then washed three times with deionized water (15 minutes per wash), embedded in 3.5% agarose (Sigma-Aldrich, CAS 9012-36-6, USA) at 40 °C, solidified, and mounted on epoxy resin stubs. Samples were sectioned at 150 µm thickness on a Vibratome Series 1000 Sectioning System (Ted Pella, Inc., Redding, CA, USA). Sections from each root were stored separately in three separate “pools” (per treatment, per species) in 0.1M tris-buffered saline solution (pH 7.4) with 0.1% sodium azide at 5 °C. The pools consisted of individual 96-well plates, one plate for each root, to record the precise position of each harvested section from the root tip.

Each of the three biological replicates mentioned in this protocol consisted of independently grown legume plants harvested on separate occasions. For each biological replicate, a minimum of 5 individual roots (technical replicates) were processed, and sections from each root were maintained and analyzed separately rather than pooled for quantitative purposes. Section “pools” were used solely as an organizational framework for storage and tracking; all imaging and interpretation were conducted at the level of individual sections linked to their originating root and biological replicate.

### 4.3 Transmission electron microscopy

Root tissue samples from non-flooded (0 hour) and 12-hour flooded timepoints were harvested from *C. arietinum*, *P. coccineus*, and *P. sativum.* Samples were sectioned into 1 mm segments and placed in 1% paraformaldehyde, 2% glutaraldehyde fixative in 0.1M sodium cacodylate buffer (pH 7.4) for 24 hours. Samples were washed three times with distilled water, then incubated in a solution of 2% osmium tetroxide and 0.1M sodium cacodylate buffer (pH 7.4) for 24 hours. Samples were washed three times in distilled water, exposed to a graded ethanol dehydration series, then placed into 1:3, 1:1, 3:1 ratio of 100% ethanol: Spurr’s resin. Samples were placed in Spurr’s Resin to cure for 48 hours, then sectioned to 20 nm thickness with an ultramicrotome. Samples were gathered onto 200 mesh copper grids and stained with Reynolds’ lead citrate (Reynolds, 1963), 0.5% uranyl acetate and 1% ruthenium red solutions. Micrographs were recorded at 20,000x and 50,000x magnification on a JEOL JEM-1200EX II TEM at 120 keV.

### 4.4 Immunolocalization

Ten sections from each species pool (five samples for non-flooded treatment, and five samples for each flooding timepoint), were placed into sterile 24-well cell culture plates and blocked with 7% normal goat serum (Thermo Fisher Scientific, USA) for 24 hours at 5 °C. For each species and time point, three biological replicates were analyzed, with five individual roots per replicate (n = 3 biological replicates; n = 5 roots per replicate). From each root, 2–3 sections were selected for imaging based on consistent anatomical position, resulting in approximately 10–15 sections imaged per biological replicate per treatment. Samples were washed 3x (15 minutes per wash) with 10 mM Tris-buffered saline (pH 7.4) containing 0.1% TWEEN-20 (TBST) then incubated with 1/20 dilutions of the following monoclonal antibodies (**Table 1**) for 24 hours at 5 °C: LM10 (rat monoclonal IgG, Plant Probes, University of Leeds, UK), CCRC-M1 (mouse monoclonal IgG1, Complex Carbohydrate Research Center, University of Georgia, USA), and CCRC-M100 (mouse monoclonal IgG1, Complex Carbohydrate Research Center, University of Georgia, USA). In addition, a carbohydrate-binding module (CBM) for crystalline cellulose – CBM3a (histidine-tagged recombinant protein CBM, Plant Probes, University of Leeds, UK) (**Table 1**) – was also utilized in the immunolocalization protocol. Samples were incubated with 1/20 dilutions of CBM3a for 24 hours at 5 °C. After incubation with monoclonal primary antibodies, samples were washed three times with TBST buffer. To achieve high host specificity and avoid target cross-talk, secondary antibodies were explicitly chosen to match the host species of the primary lines. Rat primary sections (LM10) were probed using a 1/500 dilution of cross-adsorbed IgG goat anti-rat secondary antibody conjugated to Alexa Fluor™ 647. For the mouse primary lines (CCRC-M1 and CCRC-M100), sections were successfully incubated using a 1/500 dilution of cross-adsorbed IgG goat anti-mouse secondary antibody conjugated to Alexa Fluor™ 647 (Thermo Fisher Scientific, USA) for 24 hours at 5 °C.

**Table 1:** List of primary probes and monoclonal antibodies for cell wall labeling.

Primary antibody	Isotype	Host	Antigen
CBM3a	Anti-Histidine	Mouse	Crystalline cellulose
CCRC – M1	IgG1	Mouse	Fucosylated Xyloglucan
CCRC – M100	IgM	Mouse	Non-fucosylated Xyloglucan
LM10	IgG2c	Rat	Xylan

Samples incubated with CBM3a were washed three times with TBST buffer and treated with 1/500 dilution of IgG2a mouse anti-polyHistidine secondary antibody (Sigma-Adrich, Catalog # H1029) for 24 hours at 5 °C. These samples were then washed three times with TBST buffer and treated with a 1/500 dilution of IgG goat anti-mouse secondary antibody conjugated to Alexa Fluor™ 647 fluorescent dye (Thermo Fisher Scientific, USA) for 24 hours at 5 °C.

All samples were washed a final time with three changes of TBST buffer and mounted in 100% glycerol (Sigma-Aldrich, CAS 56-81-5, USA). Negative controls used in the development of this protocol consisted of no-primary-antibody and no-CBM incubations to account for any potential non-specific epitope binding by the secondary or tertiary antibodies conjugated to Alexa Fluor™ 647. No-secondary antibody (for CCRC-M1, CCRC-M100 and LM10) or tertiary antibody (for CBM3a) incubations were performed to account for potential fluorescence emissions in the range of Alexa Fluor™ 647 (i.e. emission range ~660–720 nm). Additionally, autofluorescence-only reference controls omitting all primary and secondary complexes completely were evaluated alongside the standard preparations to precisely isolate and distinguish native, aldehyde-induced background fluorescence from probe-specific target emission tracks during confocal configuration.

### 4.5 Enzyme pretreatment assay

The enzyme pretreatment assay was modified from previous methods (Pegg et al., 2020; Xiao et al., 2016; Hernandez-Gomez et al., 2015). Root sections from the 24-hour flooding treatment timepoint of each legume species were separately incubated in 10 mL glass test tubes according to vendor instructions in the following enzyme solutions at 50 °C for 2 hours: 4% Cellulase (Sigma-Aldrich, CAS# 9012-54-8, ≥1000 units/g, suspended in 0.05 M citrate buffer), 1% xylanase (Sigma-Aldrich, CAS 37278-89-0, ≥2500 units/g, suspended in 0.05 M citrate buffer, pH 8.2), 2% pectate lyase (Megazyme, CAS# 9015-75-2, ~500 units/mg, suspended in 0.1M sodium acetate buffer, pH 5.5), 2% xyloglucanase (Megazyme, E-XEGP, CAS# 76901-10-5, ~1000 units/mL, suspended in 0.1 M sodium acetate buffer, pH 5.5), and 3% pectinase (Sigma-Aldrich, CAS# 9032-75-1, ≥2500 units/mL) in 0.05 M citrate buffer (pH 5.0). Control treatments for antibody binding (**Table 1**) entailed incubation of samples in 0.05 M citrate buffer (pH 5.0) at 50°C for 2 hours to replicate the standard primary antibody binding pattern observed without enzyme pretreatments. Incubation settings were held at 50 °C to align with manufacturer-stated optimal requirements for exogenous enzyme activity that influence the plant tissue samples selected for this assay. The incorporation of a parallel, temperature-matched control lacking active enzyme isolates (e.g., 0.05M) was used to assist in evaluating whether changes in antibody binding patterns were derived from programmatic extraction rather than artifactual shifts or structural changes to the cell wall framework induced by temperature alone. Samples were then washed three times with TBST buffer, treated with a monoclonal antibody, and incubated with secondary antibody or tertiary antibody conjugated to Alexa Fluor^®^ 647 (Thermo Fisher Scientific, USA) by the same protocol detailed in the previous *Immunolocalization* section.

### 4.6 Fluorescence microscopy

The samples from immunolocalization and enzyme pretreatment assays were observed on an Olympus FV500 Laser Scanning Confocal system (Olympus Corporation, USA) using 20x/0.70 NA and 40x/0.75 NA dry objectives. A Photometric HQ cooled CCD camera (Teledyne Photometrics, Tucson, AZ, USA) was used to capture sample images. Excitation of aldehyde-induced autofluorescence and Alexa Fluor^®^ 647 dye was achieved with 405nm and 633nm laser diodes, respectively. Default proprietary software bundled with the Olympus FV500 Laser Scanning Confocal system permitted 1024x1024 image capture via two simultaneous fluorescence channels: one corresponding to each excitation wavelength (i.e., 405nm and 633nm). Aldehyde-induced fluorescence of legume root tissue cell walls was visualized by the channel for the 405nm excitation wavelength. Fluorescent emissions indicating cell wall epitope labeling by Alexa Fluor^®^ 647 were visualized by the channel for the 633nm excitation wavelength. The average fluorescence intensity of images from each channel were recorded as a series of ~ 250μm z-stack slices that were processed via ImageJ software (NIH) and compiled single micrographs. The processed micrographs of each channel were then assigned blue LUT and red/orange LUT values to highlight aldehyde-induced fluorescence and Alexa Fluor^®^ 647, respectively, and overlaid to produce the final micrographs observed in most of the manuscript figures (**Figures 1**–**5**; **Supplementary Figure 1**–**3**). For the immunolocalization evaluation and enzyme pretreatment assay experimental protocols, a minimum of 5 processed micrographs were generated for each treatment, per species, from each experiment.

For each treatment, species, and time point, images presented in the figures are representative of consistent labeling patterns observed across biological replicates and multiple independently imaged sections. Image acquisition settings (laser power, detector gain, and offset) were held constant within each antibody experiment to enable direct visual comparison of signal patterns. Fluorescence signal evaluation was conducted qualitatively based on spatial distribution, relative presence or absence of labeling, and apparent changes in signal continuity across tissues. Although average fluorescence intensity values were recorded during image processing, these measurements were not used for formal quantitative comparison or statistical analysis due to variability in tissue structure, section thickness, and antibody penetration. As a result, no statistical tests were performed. Differences in antibody labeling were assessed through comparative visual analysis across biological replicates, focusing on reproducibility of spatial antibody labeling patterns, their tissue-specific localization, and consistent shifts in fluorescence signal presence or absence across time points and treatments.

## Data Availability

The raw data supporting the conclusions of this article will be made available by the authors, without undue reservation.
