# Supplementary figures and images for: Cell wall xyloglucan epitope modifications during flooding-induced root aerenchyma development in select cool-season legumes
Source: Front Plant Sci. 2026 Jul 8;17:1873762. doi: 10.3389/fpls.2026.1873762 (PMC13409373; doi:10.3389/fpls.2026.1873762)

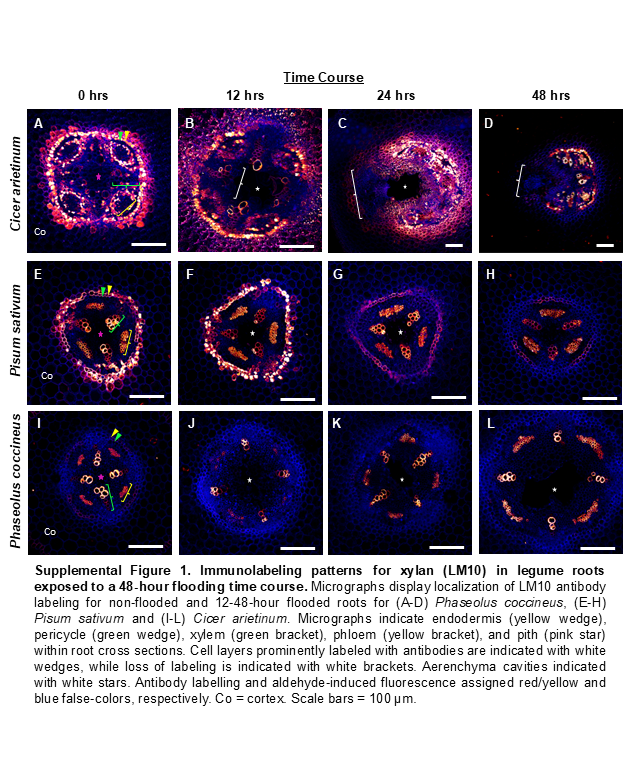

Supplement: Supplementary file 1 [file Image1.tif]

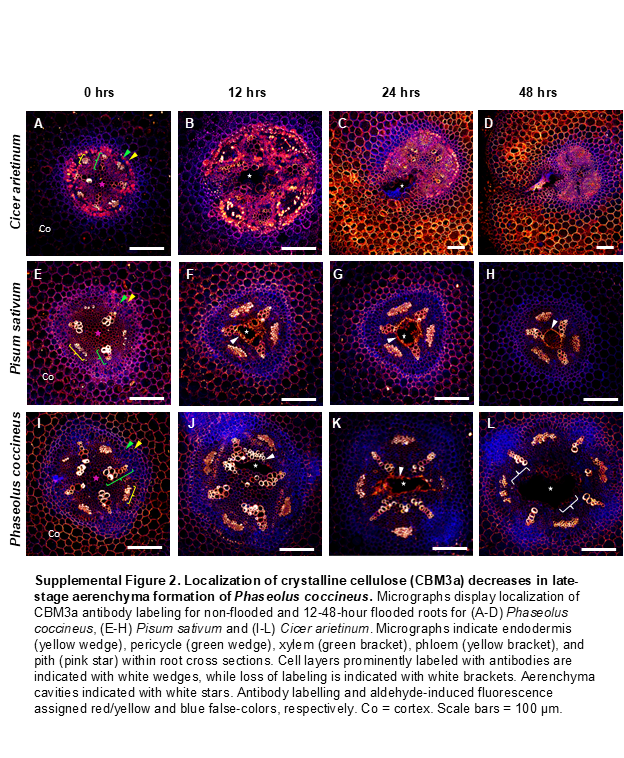

Supplement: Supplementary file 2 [file Image2.tif]

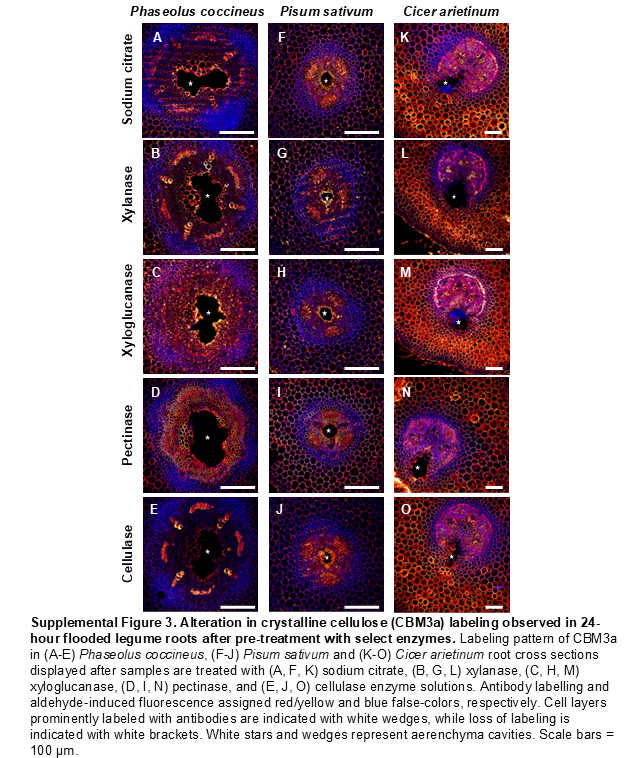

Supplement: Supplementary file 3 [file Image3.tif]
